# Dental Notes

**Published:** 1899-12

**Authors:** F. S. Casper

**Affiliations:** Austin, Texas


### Dental Department.

F. S. CASPER, D. D. S., AUSTIN, TEXAS.

### Dental Notes.

MANUFACTURERS of artificial teeth have notified all dentists that on account of the rise in platinum (?) teeth are also advanced in price But the poor dentist has been constantly lowering his prices ever since we can remember.

DENTISTS IN STATE INSTITUTIONS.—In the State of Georgia a dentist has been appointed in the State Sanitarium by the trustees. This action should receive the hearty commendation of Georgia's citizens; and other States would do well to follow the example.

WE ARE indebted to Messrs. *Chas. H. Phillips Chemical Co.* of New York City, for recent favors, in the shape of samples of their fine preparations. It is needless for us to praise the merits of their Milk of Magnesia as an antacid. We presume every dentist knows that it has no equal. And their preparation of Cocoa has only one objection, and that is, we could not get enough of it without depriving our better half and the children.

THERE is a paste prepared and placed upon the market now, which is intended to mummify the nerve of a tooth and let it remain in the roots, while a filling is placed over it. If properly done, it is claimed, that no trouble will result. This is no doubt true; but we question whether it is not best to remove the nerve outright and fill the roots, rather than to leave this "Crypt of Egyptian Darkness" as a slumbering volcano on a small scale, which is liable to burst forth at any time, and cause trouble.

---

DEATH FROM LANCING THE GUMS.—A case of death from lancing the gums of a strong, robust child, is reported in the *International Dental Journal*, November, 1899. The writer, commenting on this occurrence, says: "This accident will be the cause of untold suffering to little ones wherever this case is reported. Fond parents will dread the lancet, not thinking or knowing of the great benefit of this instrument when judiciously used, but fearing a like result." He also reports a death from the dislodging of a loose temporary tooth, and subsequent death from hemorrhage.

---

A SECOND administration of Nitrous Oxide Gas came near causing the death of a male patient, who called to have two teeth extracted. The first was extracted without trouble, but when the gas was administered the second time, it was with difficulty that the patient could be saved.

[NOTE.—Nitrous Oxide Gas is the production of the nitrate of ammonia, and although there has been only one death to seventy-five thousand cases, it is nevertheless dangerous. Injurious effects have followed its administration days and even weeks afterwards. It is especially injurious to the lungs and kidneys.]

---

THE TOOTH CROWN COMPANY.—There are "Razors in the air" for the poor dentist again. "The International Tooth Crown Company" has secured a new lease on life through a decision in a New York case, and they propose to make every dentist in practice, not only pay a royalty on all crowns or bridge work he may do, but also twenty-five dollars a year back taxes since the year 1883. Their mode of forcing the collection of the amounts claimed, was to place a United States marshal in the office of every dentist who refused to pay this demand, and in this way force the collection. "The Dental Protective Association" was the only means by which the dentists could combat this unjust monopoly. They fought the case successfully, and had the claims of the Crown Company declared null and void, but it seems they have secured a new lease of life, and it behooves the dentists to join the Protective Association, as they are not able singly to stand a suit.

